# ﻿Description of *Naviculavanseea* sp. nov. (Naviculales, Naviculaceae), a new species of diatom from the highly alkaline Lake Van (Republic of Türkiye) with complete characterisation of its organellar genomes and multigene phylogeny

**DOI:** 10.3897/phytokeys.241.118903

**Published:** 2024-04-08

**Authors:** Elif Yılmaz, David G. Mann, Romain Gastineau, Rosa Trobajo, Cüneyt Nadir Solak, Ewa Górecka, Monique Turmel, Claude Lemieux, Nesil Ertorun, Andrzej Witkowski

**Affiliations:** 1 Institute of Marine and Environmental Sciences, University of Szczecin, Mickiewicza 16A, PL70–383 Poland; 2 Royal Botanic Garden Edinburgh, Edinburgh EH3 5LR, Scotland, UK; 3 Marine and Continental Waters, Institute for Food and Agricultural Research and Technology (IRTA), Crta de Poble Nou Km 5.5, E-43540 La Ràpita, Catalunya, Spain; 4 Department of Biology, Faculty of Science & Art, Dumlupınar University, 43000 Kütahya, Türkiye; 5 Département de biochimie, de microbiologie et de bio-Informatique, Institut de Biologie Intégrative et des Systèmes, Université Laval, Québec, QC, Canada; 6 Department of Biology, Science Faculty, Eskişehir Technical University, 26000 Eskişehir, Türkiye; † Deceased

**Keywords:** Group I intron, LAGLIDADG, mitogenome, Naviculaceae, plastome, pseudogene, soda lake

## Abstract

The current article describes *Naviculavanseea***sp. nov.**, a new species of diatom from Lake Van, a highly alkaline lake in Eastern Anatolia (Türkiye). The description is based on light and scanning electron microscopy performed on two monoclonal cultures. The complete nuclear rRNA clusters and plastid genomes have been sequenced for these two strains and the complete mitogenome for one of them. The plastome of both strains shows the probable loss of a functional *ycf35* gene. They also exhibit two IB4 group I introns in their *rrl*, each encoding for a putative LAGLIDADG homing endonuclease, with the first L1917 IB4 intron reported amongst diatoms. The Maximum Likelihood phylogeny inferred from a concatenated alignment of *18S*, *rbcL* and *psbC* distinguishes *N.vanseea* sp. nov. from the morphologically similar species *Naviculacincta* and *Naviculamicrodigitoradiata*.

## ﻿Introduction

Lake Van is located in Eastern Anatolia, Turkey (Republic of Türkiye). It is Turkey’s largest inland water body and also world’s largest soda lake. The lake is surrounded by dormant volcanoes and its formation was a consequence of the eruption of the Nemrut stratovolcano (not to be confused with the Nemrut Mountain, also in Turkey), which is 2247 m above sea level. As a result of the erosion of volcanic rocks in the catchment and evaporation, the lake water is salty (21.4‰) and alkaline (155 m mEq–1, pH 9.81) (Glombitza et al. 2013; Ersoy Omeroglu et al. 2021). The lake is notable for its unusual chemistry, which results from the constant losses of calcium as carbonate and of magnesium in the form of mineral phases rich in Mg–silica. Thus, the Mg cycle is closely related to the silica cycle, which is itself dependent on the production of biosilica by diatoms, eventually followed by the dissolution of their frustules (Reimer et al. 2009).

The genus *Navicula* is amongst the most species-rich genera of Bacillariophyceae, although this is partly because it was used for a long time as a ‘catch-all’ for simply structured, bilaterally symmetrical raphid diatoms. It was erected as early as 1822 by Bory in his ‘Dictionnaire Classique d’Histoire Naturelle’ (Bory de Saint-Vincent 1822). The name chosen by Bory refers to the shape of the cells, similar to the shuttle that was used for weaving. The cells are generally solitary and motile, although some species live in mucilage tubes (Millie and Wee 1981). Cells have two parietal chloroplasts. Their valves are symmetrical both apically and transapically and have rounded, acute or capitate ends. The central area is often distinctly expanded (Patrick 1959; Cox 1979; Round et al. 1990).

The only account ever published on the diatoms from Lake Van was written by Legler and Krasske (1940). Amongst the 24 species they recorded were three taxa of *Navicula*, all of them considered as varieties of *Naviculacryptocephala*, namely *Naviculacryptocephala* Kützing, 1844, Naviculacryptocephalavar.intermedia Grunow 1880 and a taxon noted as Naviculacryptocephalavar.veneta (Kützing) Grunow, which possibly corresponds to Naviculacryptocephalavar.veneta (Kützing) Rabenhorst, 1864. Out of these three taxa, only *N.cryptocephala* Kützing, 1844 is still deemed to be valid. Naviculacryptocephalavar.intermedia is considered a synonym of *Naviculacapitatoradiata* H. Germain ex Gasse 1986 and Naviculacryptocephalavar.veneta is now treated as an independent species, *Naviculaveneta* Kützing 1844.

*Navicula* species are rather well documented in inland waters where they are known for their bioindicator potential (Lange-Bertalot 2001). For instance, *Naviculatripunctata* (O.F. Müller) Bory is a good indicator of eutrophic waters with an average to high electrolyte content, while taxa such as *Naviculagregaria* Donkin, 1861, *Naviculameulemansii* A.Mertens, A.Witkowski & Lange-Bertalot 2013 and *N.veneta* are common in brackish to electrolyte rich waters (Cox 1995; Adrienne Mertens et al. 2014).

Preliminary results from a new sampling campaign conducted in 2021 in Lake Van strongly suggested that the biodiversity of diatoms had been underestimated in the previous work of Legler and Krasske (1940). One illustration is a previously undocumented *Nitzschia*, *N.anatoliensis* Górecka, Gastineau & Solak (Solak et al. 2021), which would have probably been overlooked if it had not been for the combined use of microscopic and molecular tools. Amongst several other monoclonal cultures from the 2021 campaign, two contained strains of a *Navicula* species were identified, which is the subject of the present article. Although both strains were noticeably different in size, they were quickly proven to belong to the same new species.

The aim of the following article is to formally describe *Naviculavanseea* sp. nov. from Lake Van. The description combines the use of light microscopy (live specimen and cleaned frustules) and scanning electron microscopy. The complete cluster of nuclear ribosomal RNA genes and the complete plastid genome were obtained for both strains by means of next generation sequencing and also the mitogenome of one of these strains. These results were included in a multigene Maximum Likelihood phylogeny which unambiguously separated *Naviculavanseea* sp. nov. from morphologically similar known species, whose differences with *Naviculavanseea* sp. nov. are discussed. As it was the first time that a L1917 group I intron with its putative LAGLIDADG homing endonuclease gene had been discovered in the plastid genome of a diatom, special attention has been paid to this feature, with a phylogeny of the putative LAGLIDADG protein being performed.

## ﻿Materials and methods

### ﻿Sampling, isolation and cultivation

**Figure 1.**
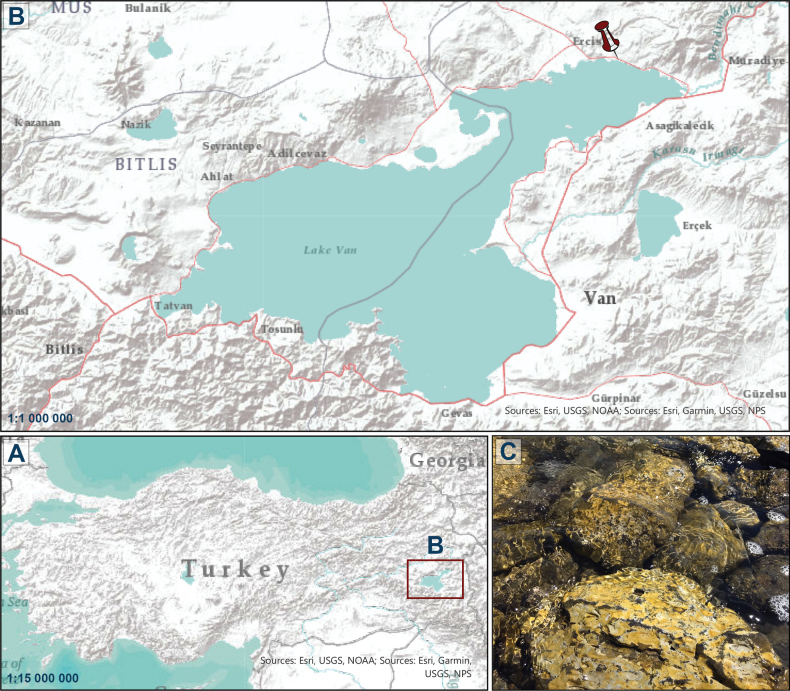
Map of the sampling location **A** location of Lake Van in Turkey. The red frame indicates the position of Lake Van **B** general view of the lake. The pin indicates the position of the sampling area **C** photo of the epilithic sampling area on the rock (Esri. (2023). ArcGIS Pro 3.1.0. Environmental Systems Research Institute).

Epilithic samples were collected by brushing rocks in the littoral of Lake Van in July 2021, in the vicinity of Erciş Municipality (Fig. 1). Samples were re-suspended in surface water from the lake in 50 ml tubes before being brought to the University of Szczecin for subsequent analyses. Samples were then transferred into Petri dishes containing sterile f/2 medium (Guillard 1975) modified to 18‰ salinity. Single cells were isolated by micropipette under an inverted Nikon Eclipse light microscope. Successive re-isolations were performed (at least 3 times) before the culture was considered monoclonal. Strains were later transferred into 250 ml Erlenmeyer ﬂasks with modified f/2 medium. Cultures were maintained in active growth under a light intensity of 60 µmol photons m^–2^ s^–1^ and a photoperiod of 14 h light/10 h darkness). Two morphologically distinct clones with different cell sizes were registered in the Szczecin Culture Collection as SZCZEY2172 and SZCZEY2262.

### ﻿Light and scanning electron microscopy

Pictures of living diatoms were taken using a Light Microscope (LM) Zeiss Axio Scope A1 (Carl Zeiss, Jena, Germany at a magnification of 1000× by transferring diatom cultures directly on to the glass slide.

To prepare cleaned frustules for microscopy, 5 ml of monoclonal cultures were transferred into 20 ml beakers with 10 ml of 10% hydrochloric acid (HCl). After 24h, samples were washed four times with distilled water then re-suspended in 30% hydrogen peroxide (H_2_O_2_) and boiled for about four hours. Finally, samples were washed again four times with distilled water. For LM, cleaned material was then air-dried on cover glasses and mounted on glass slide with Naphrax® (Brunel Microscopes Ltd., Chippenham, UK) solution and pictures were taken with the Zeiss Axio Scope A1. For SEM, a drop of cleaned sample was deposited on a Nuclepore Track-Etch membrane from Whatman (Maidstone, England). The membranes were air-dried overnight, mounted on aluminium stubs with carbon tape and coated with gold using a Q150T coater from Quorum Technologies (Laughton, U.K.). SEM observations were made at the Faculty of Chemical Technology and Engineering, Western Pomeranian University of Technology in Szczecin (Poland), using a Hitachi SU8020 (Tokyo, Japan) and Eskişehir Technical University (Türkiye) using a ZEISS Ultra microscope (Oberkochen, Germany).

### ﻿Next generation sequencing and bioinformatic analyses

DNA was extracted from clones SZCZEY2172 and SZCZEY2262 using the protocol of Doyle and Doyle (1990). Total DNA was then sent to the Beijing Genomics Institute (BGI) in Shenzhen (China) to be sequenced on a DNBSEQ platform. For each clone, a total of ca. 40M clean 150 bp paired-end reads was obtained. Reads were assembled with a k-mer parameter of 125 using SPAdes 3.15.0 (Bankevich et al. 2012). Contigs of interest were retrieved by customised command-line BLASTn analyses as previously described (Dąbek et al. 2022; Gastineau et al. 2022). Consed (Gordon and Green 2013) was used to merge the different subunits of the plastome and when trying to circularise the mitogenome. Annotations were performed using the same tools as described in Gastineau et al. (2022). The maps of the organellar genomes were obtained from the OGDRAW online portal (Lohse et al. 2013). The different parts of the nuclear rRNA gene cluster were identified with the help of Rfam 14 (Kalvari et al. 2021).

### ﻿Molecular phylogeny

The three gene datasets (*18S*, *rbcL* and *psbC*) already used in previous publications (Dąbek et al. 2017; Li et al. 2020; Górecka et al. 2021a;) were obtained and the corresponding genes from various Naviculaceae and *N.vanseea sp. nov*. appended. Sequences for *N.capitatoradiata*, *N.microdigitoradiata* Lange-Bertalot, 1993 and *N.cincta* (Ehrenberg) Ralfs 1861 were also added. However, it should be noted that these three species were represented in GenBank just by *18S* and *rbcL* (*N.capitatoradiata* and *N.cincta*) or *rbcL* only (*N.microdigitoradiata*). Genes were aligned separately with MAFFT 7 (Katoh and Standley 2013) with the -auto option and trimmed using trimAl (Capella-Gutiérrez et al. 2009) with the -automated1 option. The best model of evolution for each of these genes was selected with ModelTest-NG (Darriba et al. 2020) and were GTR+I+G4 (*psbC*), TIM3+I+G4 (*rbcL*) and TIM1+I+G4 (*18S*). Alignments were then concatenated using Phyutility 2.7.1 (Smith and Dunn 2008) for a final size of 3301 bp. A Maximum Likelihood phylogeny was constructed from the concatenated alignment using IQ-TREE 2.2.0 (Minh et al. 2020) with 1000 ultrafast bootstrap replicates and a dataset partitioned, based on the best models of evolution found for each gene. *Triparmapacifica* (Guillou & Chrétiennot-Dinet) Ichinomiya & Lopes dos Santos, 2016 was used as an outgroup.

For the phylogeny of the putative LAGLIDADG endonuclease proteins, protein sequences found in IB4 - L1917 and - L1931 introns presented in Lucas et al. (2001) and Haugen and Bhattacharya (2004) were downloaded from GenBank. Sequences from the current study, from Turmel et al. (2002), Haugen et al. (2007) and Lemieux et al. (2014) were appended. The IB2 – L1917 LAGLIDADG sequence from *Coxiellaburnetii* (Derrick, 1939) Philip, 1948 was used as an outgroup (Raghavan et al. 2007). The phylogeny was conducted in a similar way to the multigene phylogeny, except that alignment was not trimmed. The best model of evolution returned by ModelTest-NG was LG+G4.

## ﻿Results

### ﻿Taxonomy

#### ﻿ Navicula vanseea

Taxon classificationPlantaeNaviculalesNaviculaceae

Yilmaz, Gastineau, Solak & Witkowski sp. nov.

915AD59D-E312-50A1-B37D-AA72AC865D3D

Figs 2 , 3 , 4

##### Type material.

**Figure 2.**
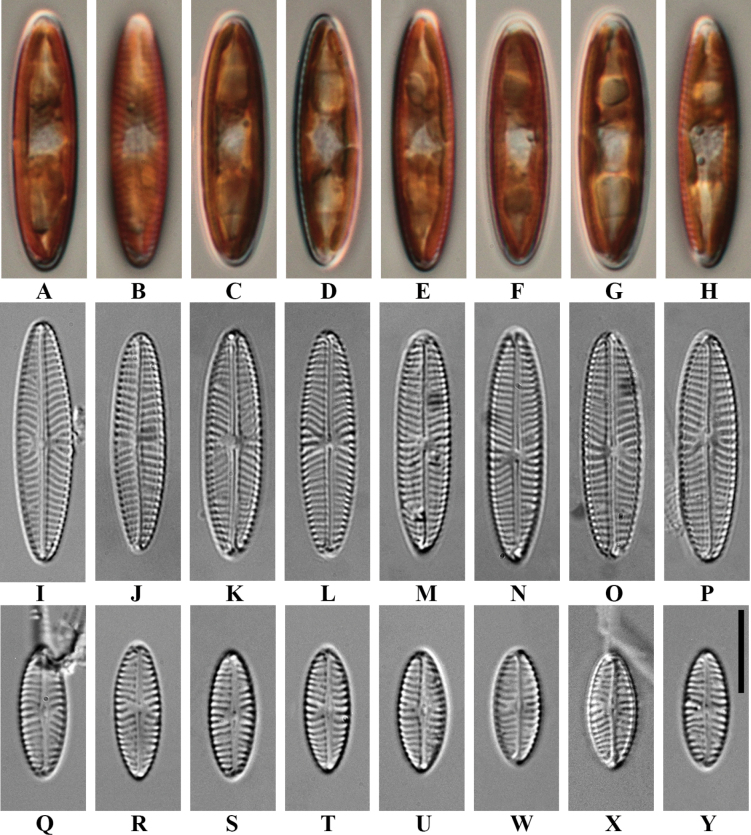
*Naviculavanseea* sp. nov. LM micrographs **A–H***in vivo* pictures of *Naviculavanseea* sp. nov. SZCZEY2172 **I**LM image of a cleaned valve from wild material **J–P** cleaned valves of *Naviculavanseea* sp. nov. SZCZEY2172 **Q–Y** cleaned valves of *Naviculavanseea* sp. nov. SZCZEY2262 Scale bar: 10 μm.

***Holotype***: Slide number SZCZEY2172 in the collection of Andrzej Witkowski at the University of Szczecin, Poland. Valves representing the holotype population are illustrated in Fig. 2L.

***Isotype***: Slide number TR_Erciş_Van_2021 deposited in Kütahya Dumlupınar University (Turkey).

##### Registration.

http://phycobank.org/104542

##### Type locality.

Erciş Van, Türkiye (38°59'47.3"N 43°24'15.3"E) collected by: Elif Yilmaz, 31 July 2021.

##### Etymology.

The name given to the species refers to the German name of Lake Van (Vansee, the sea of Van) as it was used in the work of Legler and Krasske and is meant as a tribute to these authors and their work.

##### Distribution and ecology.

The taxon was exclusively observed within benthic epilithic assemblages in Lake Van (salinity 21.4‰ and pH 9.5).

##### Description.

***LM*** (Fig. 2A–Y) Valves: smaller specimens elliptic, tapering towards cuneately rounded apices, larger specimens linear-elliptic-lanceolate narrowly rounded, with narrowly rounded poles, which are occasionally slightly protracted (Fig. 2E, K, N). Valve dimensions (n = 39): length 11.0–28.0 μm, width 5.0–6.5 μm. Raphe filiform, straight. Central area small and rounded, axial area narrow. Striae strongly radiate, sometimes irregularly shortened around the central area, 12–13 in 10 μm, lineolae difficult to resolve in LM, ca. 50 in 10 μm.

**Figure 3.**
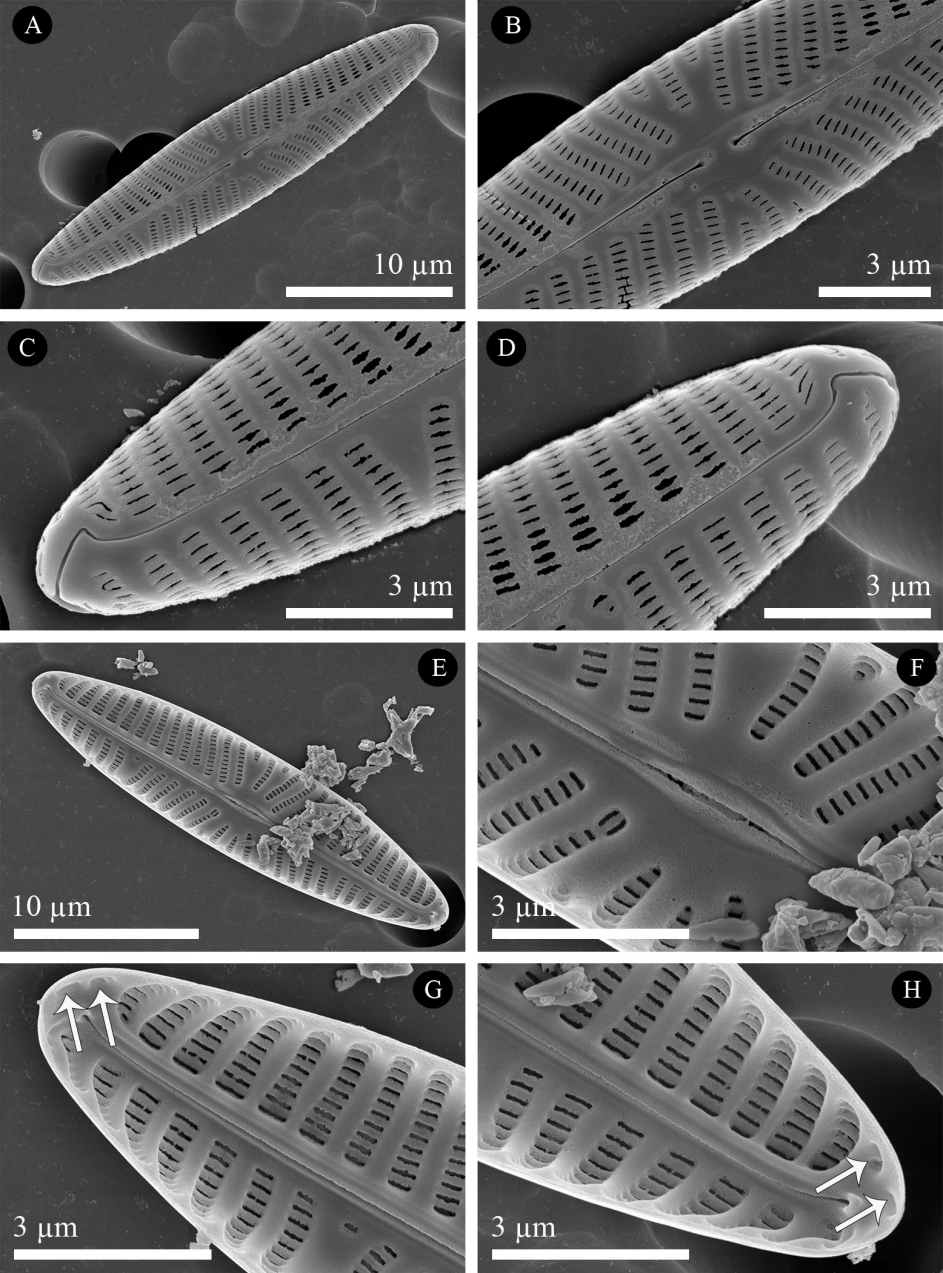
SEM micrographs of *Naviculavanseea* sp. nov. SZCZEY2172 **A** external view of the entire valve **B** details of central area showing simple, slightly drop-shaped proximal raphe endings and shortened striae **C, D** details of the two apices of a single valve showing the terminal fissures **E** internal view of the entire valve **F** details of central area showing filiform proximal raphe endings in a fusiform expansion of the raphe-sternum **G, H** details of apices showing well-developed helictoglossae showing two isolated lineolae (white arrows). Scale bars: 10 μm (**A, E**); 3 μm (**B–D, F–H**).

**Figure 4.**
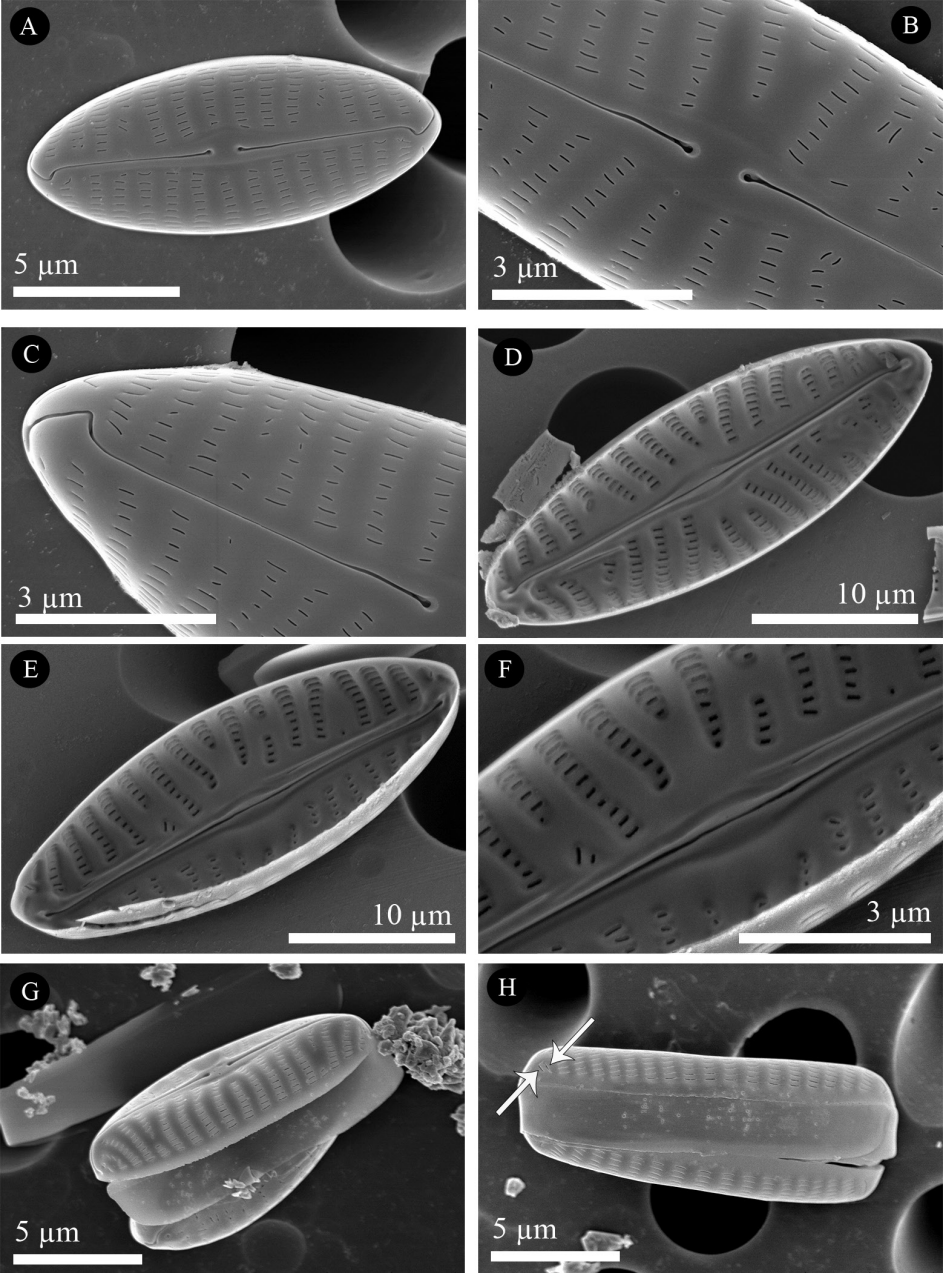
SEM micrographs of *Naviculavanseea* sp. nov. SZCZEY2262 **A** external view of the entire valve **B** details of central area showing simple proximal raphe endings and shortened striae **C** details of apex showing the terminal fissure **D, E** internal view of two entire valves, showing the central area and filiform proximal raphe endings **F** details of apex showing well-developed helictoglossae **G, H** girdle view of valves showing continuous areolation on mantle and two isolated lineolae (white arrows). Scale bars: 5 μm (**A, D, E, G, H**); 3 μm (**B, C, F**).

***SEM External valve surface*** (Figs 3A–D, 4D–F): Valve surface flat (Fig. 3G, H), areolae apically elongated (Figs 3B–D, 4B). Raphe sternum slightly elevated above the valve face level (Fig. 4G). Axial area very narrow, central area very slightly expanded, small, asymmetric (Figs 3B, 4A, B). Proximal raphe endings drop-like, slightly deflected unilaterally (Fig. 4B). Distal raphe endings strongly hooked in the same direction (Fig. 3C, D, which are the two ends of the same valve and Fig. 4A).

***SEM Internal valve surface*** (Figs 3E–H, 4D–F): valve surface slightly arched with transapical striae positioned in relatively deep grooves, bordered by virgae that become thicker towards to the centre of the valve (Figs 3H, 4D, F). Central area asymmetric, usually only slightly expanded (Figs 3E, F, 4F), but sometimes more strongly (Fig. 4D). The internal lineolae openings are slit-like (Fig. 3F–H), narrower than the vimines. Lineolae occluded by hymens (Fig. 4F); two isolated lineolae are present at the valve apex. Raphe sternum slightly widened at the centre to form a fusiform ridge enclosing the central raphe endings, which are simple, straight and separated (Figs 3F, 4F). Distally, the raphe terminates in well-developed helictoglossae (Figs 3G, H).

### ﻿Genomics and phylogeny

#### ﻿The nuclear rRNA gene cluster

The complete rRNA gene cluster was sequenced for both clones and deposited in NCBI GenBank with accession numbers OR797294 (SZCZEY2172) and OR797293 (SZCZEY2262). The cluster is 4902 bp long, distributed as follows: 18S – 1792 bp, ITS1 – 195 bp, 5.8S – 155 bp, ITS2 – 260 bp, 28S – 2500 bp. Comparing the two clones, there was one single nucleotide polymorphism (SNP) found in the 18S (in the V2 region), three in the ITS1, one in the 5.8S, three in the ITS2 and two in the 28S (both in the D1/D2 region).

#### ﻿Mitochondrial genome

**Figure 5.**
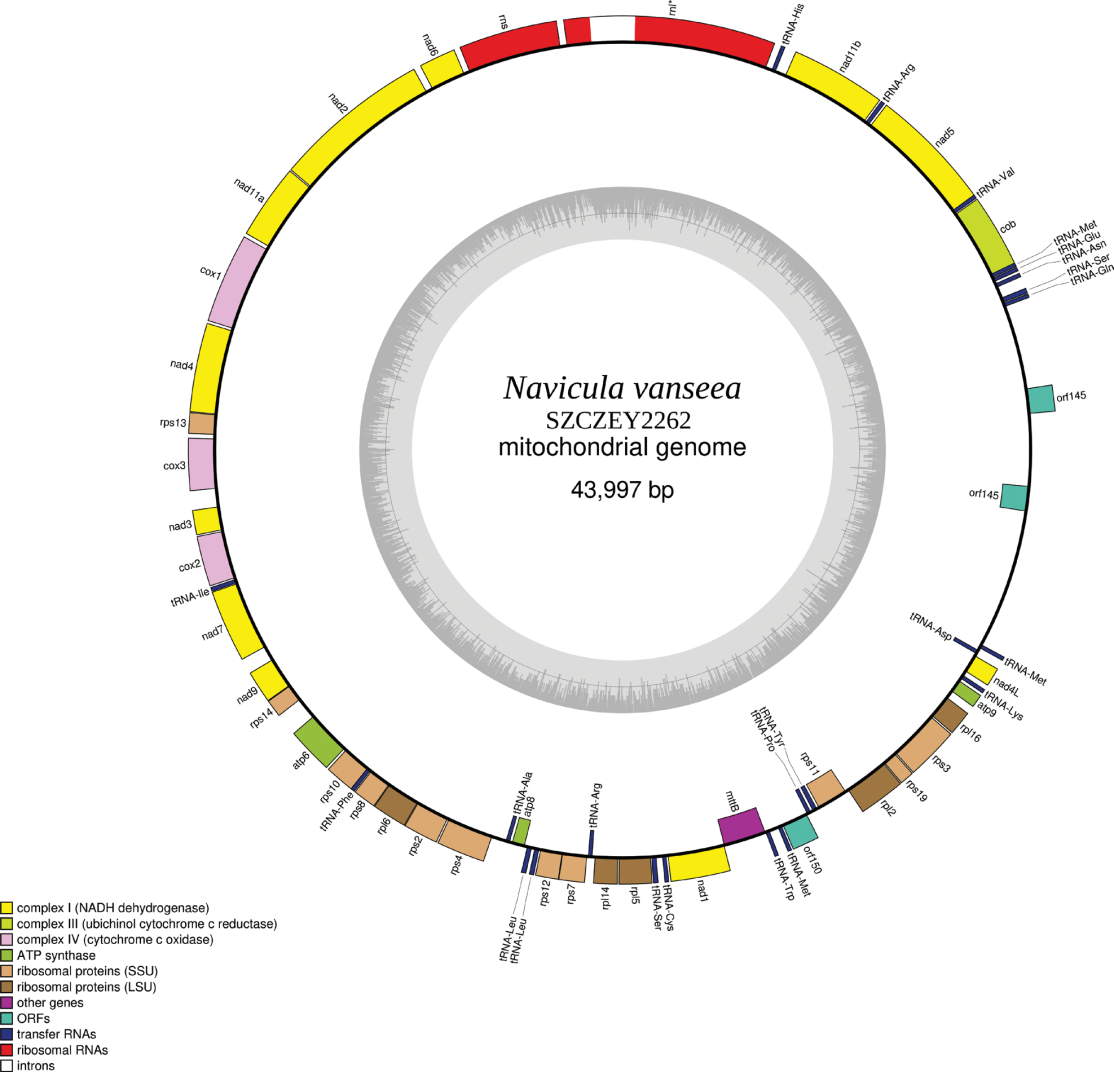
Map of the mitochondrial genome of *Naviculavanseea* sp. nov. SZCZEY2262.

A 43997 bp contig corresponding to the mitochondrial genome was retrieved for strain SZCZEY2262, but could not be circularised because of the presence of repeated sequences at its ends. However, for easier reading, it is displayed as circular on the map (Fig. 5). The mitogenome encodes 34 protein-coding genes plus the conserved open-reading frame (ORF) *orf150* between *rps11* and *mttB* (Pogoda et al. 2019), two rRNA genes and 23 tRNA genes (GenBank: OR795084). The *nad11* gene is split into two distinct subunits, separated from each other by two protein-coding genes, two rRNA and one tRNA. In the repeated part of the genome, there are two copies of the same ORF, *orf145*. There is a 767-bp group I intron in the *rnl* gene.

Despite several attempts, it was impossible to assemble the mitogenome of strain SZCZEY2172. Lowering the k-mer parameter to 75 only allowed the recovery of a short ca. 500 bp fragment with a low coverage. This fragment was used as a seed to try an assembly with NOVOPlasty 4.3.3 (Dierckxsens et al. 2017), using the mitogenome of SZCZEY2262 as reference sequence and a k-mer of 25, but this attempt also failed.

#### ﻿Plastid genome

**Figure 6.**
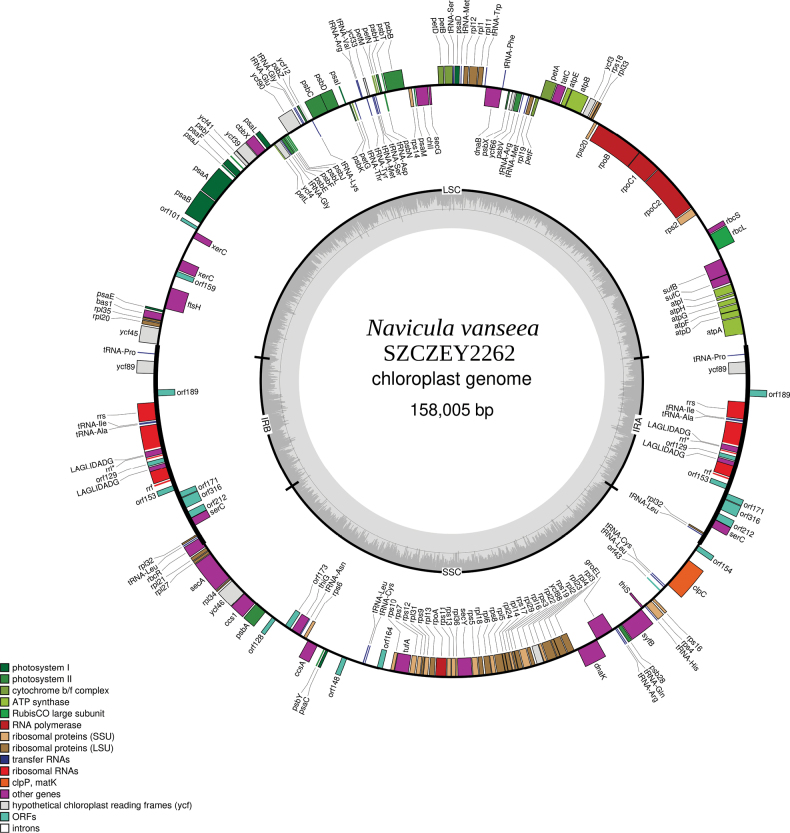
Map of the plastid genome of *Naviculavanseea* sp. nov. SZCZEY2262.

**Figure 7.**
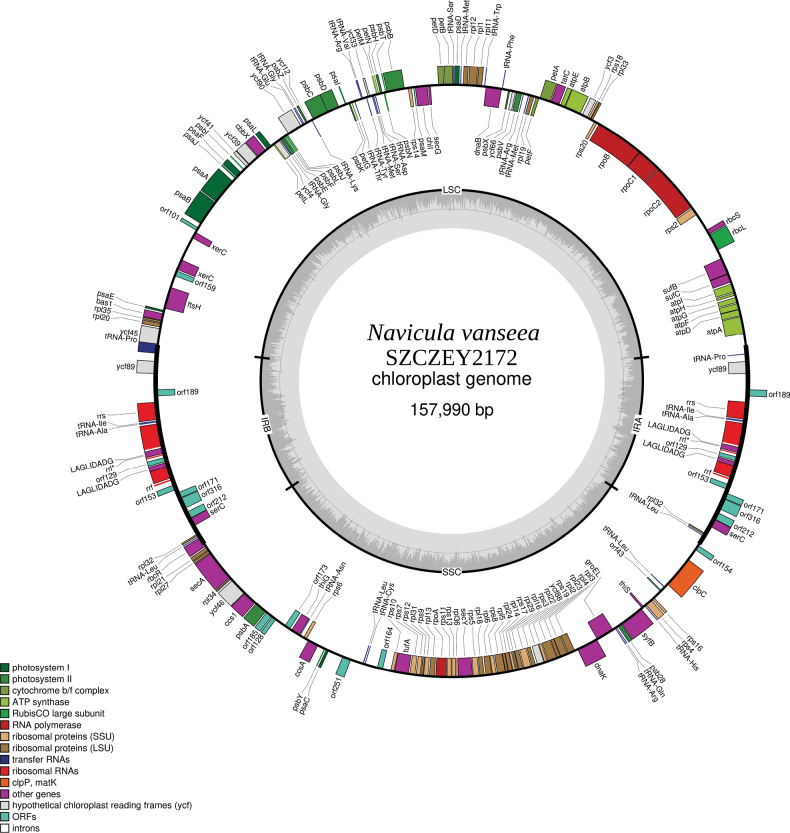
Map of the plastid genome of *Naviculavanseea* sp. nov. SZCZEY2172.

The plastome is 158,005 bp long for SZCZEY2262 (Fig. 6) and 157,990 bp long for SZCZEY2172 (Fig. 7). For SZCZEY2262 (GenBank: OR795085), the large single-copy (LSC) is 72,941 bp long and encodes 74 conserved protein-coding genes, two non-conserved ORF, two putative integrase/recombinase *xerC* genes and 17 tRNAs. The small single-copy region (SSC) is 49,714 long and encodes 51 conserved protein-coding genes, eight tRNAs, and five non-conserved ORFs of a size higher than 100 amino-acids (AA). The inverted repeat (IR) is 17,675 bp long and encodes one conserved protein-coding gene, three rRNAs, six non-conserved ORFs, four tRNAs and one putative *serC* gene and *rbcR* overlaps the inverted repeat B (IRB) and the SSC. There are two IB4 group I introns in the *rrl* gene at positions 1917 and 1931 (based on the reference sequence U00096 from *Escherichiacoli* T. Escherich, 1885 str. K-12 substr. MG1655), both containing two putative *LAGLIDADG* homing endonuclease genes. They will be referred to as L1917 and L1931.

For SZCZEY2172 (GenBank: OR795086), the LSC is 72,913 bp long and has an identical gene content compared to SZCZEY2262, the SSC is 49727 bp long and encodes 51 conserved protein-coding genes, eight tRNAs and six non-conserved ORFs of more than 100 AA. The IR is 17,675 bp long and has an identical gene and intron content to SZCZEY2262, with the same overlap of *rbcR* between the IRB and the SSC.

Both genomes contain a 43 AAORF in their SSC that cannot be extended because of the presence of stop-codons. This ORF shows similarities to the hypothetical chloroplast RF35 encoded by *ycf35*, which is missing between both strains. The position of this ORF also corresponds to the position of *ycf35* in *Naviculaveneta*, between *clpC* and *rps13* (Gastineau et al. 2021a).

It is worth noting that, in addition to the differences in length and content in the non-conserved ORFs, there is a slight degree of extra polymorphism in the two strains, the extent depending on the part of the genome considered. There were only two SNPs in the inverted repeat (one in the spacer between *ycf45* and *tRNA-Pro* and the other inside *rbcR*). On the other hand, a gene such as *psbC* displayed three SNPs, two of them silent, but one leading to a phenylalanine–leucine substitution. The two *xerC* genes, although present in both strains, differed in length.

#### ﻿Multigene phylogeny

**Figure 8.**
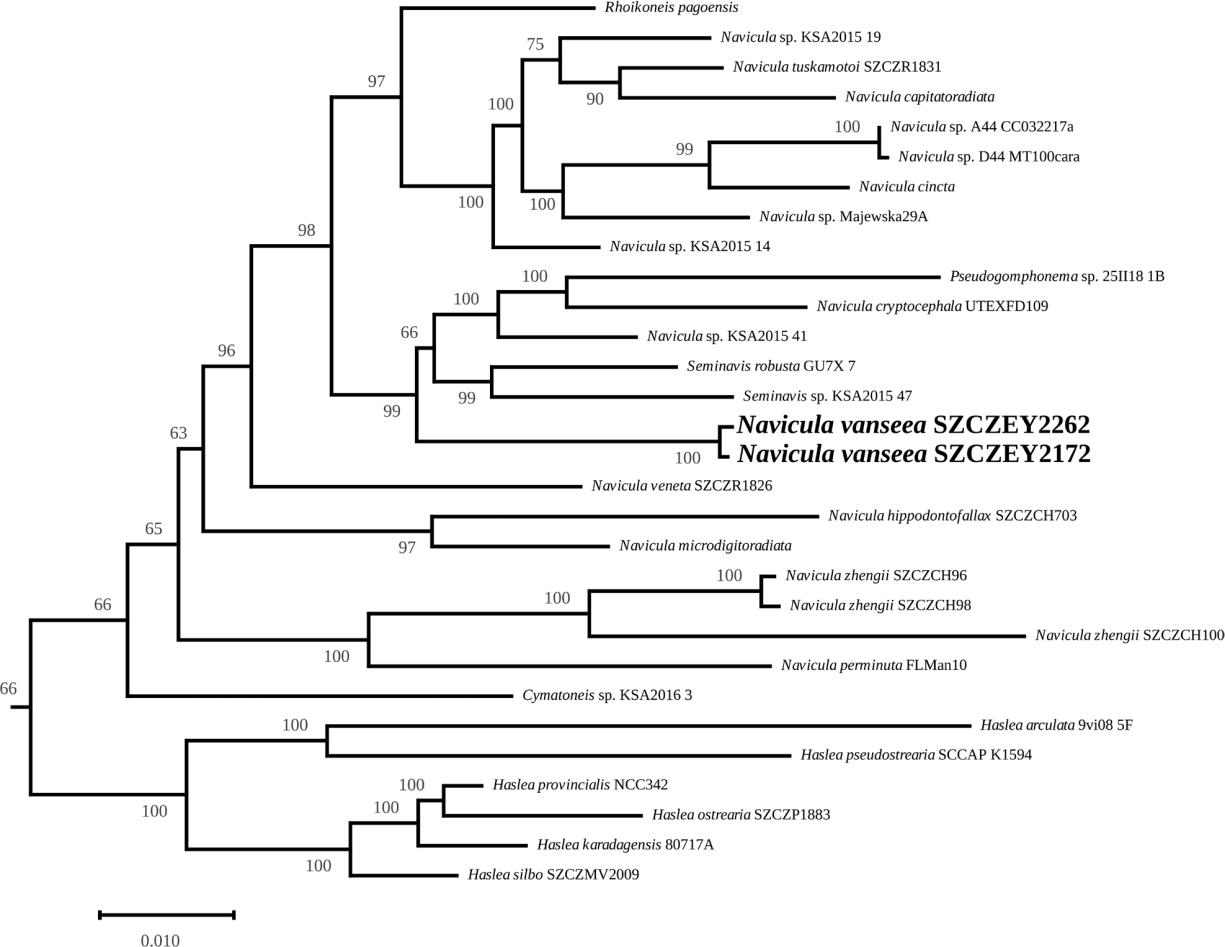
Maximum Likelihood phylogenetic tree obtained from concatenated alignments of *psbC*, *rbcL* and *18S*.

The subtree containing Naviculaceae (Fig. 8) has been extracted from the complete multigene tree (downloadable as explained in the data availability statement). *Naviculavanseea* sp. nov. appears in a well-supported (99%) clade that also contains the freshwater diatom *Naviculacryptocephala* UTEX FD109 (sometimes indexed as *Naviculacryptocephala var. veneta* UTEX FD109), a specimen isolated by the late David B. Czarnecki from North Dakota, USA (Theriot et al. 2010) and the marine *Navicula* sp. KSA2015 41, which originates from the vicinity of Rabigh on the Red Sea, Saudi Arabia (Sabir et al. 2018). The clade also contains *Pseudogomphonema* sp. and two species of *Seminavis* spp. The phylogeny unambiguously separates *N.vansee* sp. nov. from the two morphologically similar species *N.cincta* and *N.microdigitoradiata. Naviculacincta* appears in a different, strongly-supported clade (97%) that contains *N.capitatoradiata*, *Naviculatsukamotoi* (Sterrenburg & Hinz) Yuhang Li & Kuidong Xi 2017, several unnamed species of *Navicula* spp. and *Rhoikoneispagoensis* Lobban, 2015. *Naviculamicrodigitoradiata* is also easily distinguished and appears as sister to *Naviculahippodontofallax* Witkowski & Chulian Li 2016.

#### ﻿Phylogeny of the putative homing endonuclease LAGLIDADG proteins

**Figure 9.**
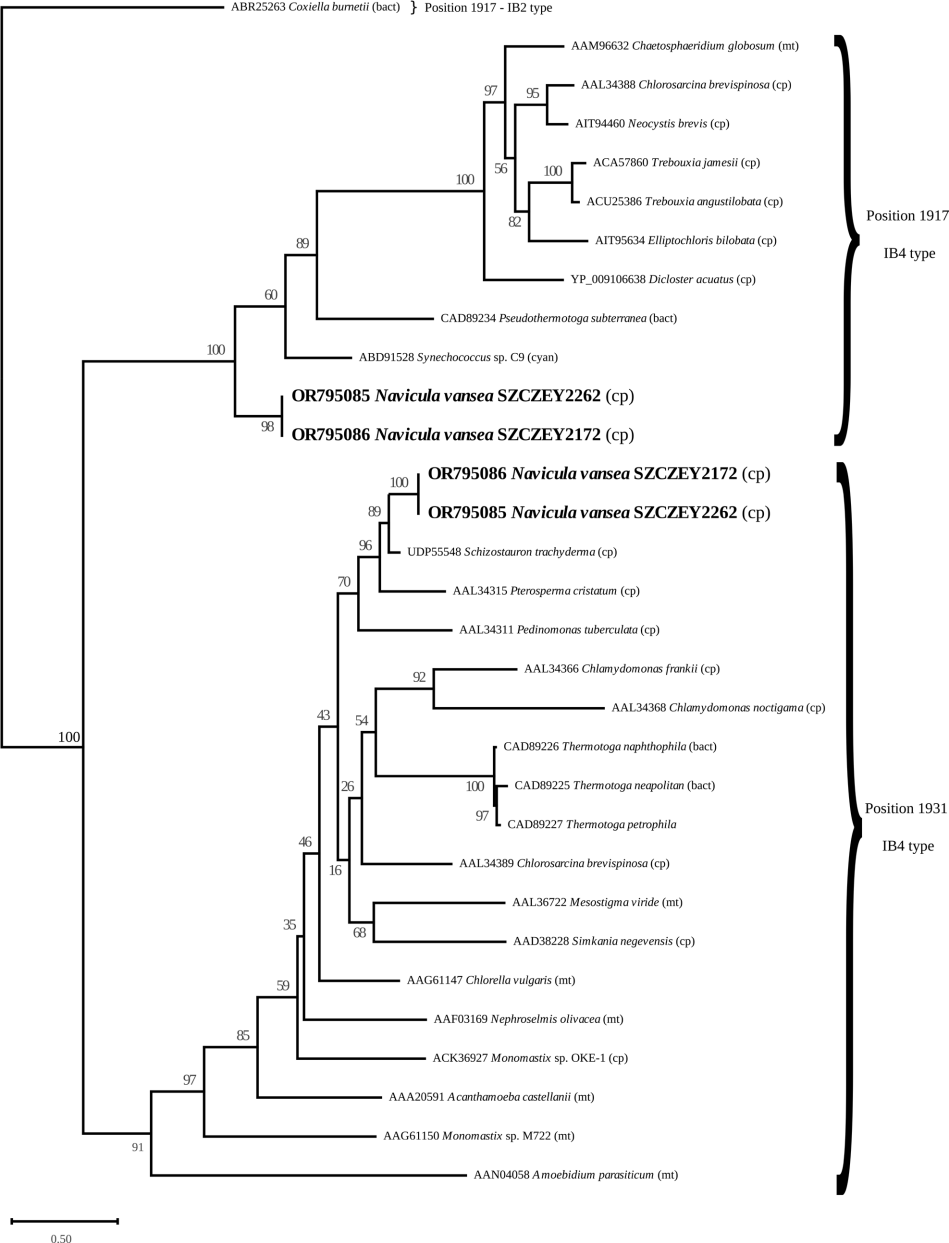
Maximum Likelihood phylogenetic tree inferred from the alignment of the putative LAGLIDADG endonuclease proteins found in the group I introns of *Naviculavanseea* sp. nov. and other taxa. The type of genome is indicated between brackets: cp – plastome, mt – mitogenome, bact – bacteria, cyan – cyanobacteria.

Once rooted with sequence ABR25263, the phylogenetic tree of LAGLIDADG proteins (Fig. 9) distinguished the two groups. The tree associates sequences from *N.vanseea* SZCZEY2172 and SZCZEY2262 with those in other species that are of the same type and occupy the same positions. For example, the L1931 LAGLIDADG sequences of the two *N.vanseea* clones were found to be sister to a L1931 LAGLIDADG in the plastid genome of the diatom *Schizostaurontrachyderma* (F. Meister) Górecka, Riaux-Gobin & Witkowski, 2021 (Górecka et al. 2021b) and then to the green algae *Pterospermacristatum* Schiller, 1925 (Prasinophyceae) and *Pedinomonastuberculata* (Vischer) Gams, 1947 (Pedinophyceae), a synonym of *Chlorochytridiontuberculatum* Vischer 1945 (both from plastid genomes). In contrast to the topology of the L1931 clade, in the L1917 tree, the *N.vanseea* LAGLIDADG sequences appeared at the base of the clade with maximum support. In this clade, sequences from the plastomes of various Viridiplantae form a strong clade, separated from *N.vanseea* by two Prokaryota, namely the heterotrophic bacteria *Pseudothermotogathermarum* (Windberger et al. 1992) Bhandari and Gupta 2014 and the cyanobacterium *Synechococcus* sp. C9.

## ﻿Discussion

### ﻿Morphological comparison with similar taxa

*Naviculavanseea* sp. nov. has elliptic valves that taper towards cuneately rounded apices in smaller specimens and linear-elliptic-lanceolate with narrowly rounded, protracted endings in larger specimens (Table 1). *Naviculacincta*, *Naviculadealpina* Lange-Bert., *N.microdigitoradiata*, *N.meulemansii* and *Naviculaveronensis* Lange-Bertalot and Cantonati are the most similar taxa. Regarding valve outline, *Naviculadealpina* is very similar, being linear-lanceolate to almost elliptic. However, the apices of *N.dealpina* are much narrower compared to the bluntly rounded apices of *N.vanseea*. Moreover, *N.dealpina* is larger (26–86 µm length, 8–12 µm width) and has a lower stria density (8–10 striae in 10 µm) than *N.vanseea* (11–28 µm length, 5–6 µm width and 12–13 striae in 10 µm) and it has a transversely rectangular central area (not elliptic and small in *N.vanseea*). Regarding dimensions, *N.veronensis* and *N.meulemansii* are quite similar. *Naviculaveronensis* has similarly linear-lanceolate valve outline, but *N.vanseea* is more elliptic. Moreover, *N.veronensis* has relatively larger and more visible central area with gradually wedge-shaped, finally obtusely rounded apices, while *N.vanseea* has an indistinctive central area with narrowly rounded apices. In SEM, *N.veronensis* has a thicker sternum structure than *N.vanseea*, while *N.vanseea* has more strongly-hooked distal raphe endings. Similarly, *N.meulemansii* have thicker sternum structure in SEM. Additionally, *N.meulemansii* has elliptic-lanceolate valve outline with cuneate apices and higher striae density than *N.vanseea* (15–21 in 10 µm vs. 12–13 in 10 µm, respectively). Amongst the similar taxa, *Naviculamicrodigitoradiata* and *N.cincta* have slightly larger sizes (up to 40–45 µm length and 7–8 µm width) and lower striae density (max. 10 in 10 µm for *N.cincta* and 11 in 10 µm for *N.microdigitoradiata*) than *N.vanseea* (max. 28 µm length, 6 µm width and 13 striae in 10 µm). *N.meulemansii* has an almost similar length with narrower specimens (3–5 µm width) and higher striae density (15–21 striae in 10 µm). The valve outline is relatively more elliptic with cuneately rounded apices in *N.meulemansii*, while *N.vanseea* has a more linear-lanceolate outline with narrowly-rounded apices (Table 1). It is worth mentioning that *N.cincta*, *N.veronensis* and *N.dealpina* are also all species suspected to prefer alkaline water (Cantonati et al. 2016), so it is especially important to clarify the distinctions between them and *N.vanseea*.

**Table 1.** Comparison of *Naviculavanseea* sp. nov. and similar species.

	*Naviculavanseea* sp. nov.	*Naviculameulemansii*	*Naviculamicrodigitoradiata*	*Naviculacincta*	*Naviculacariocincta*	*Naviculaveronensis*	*Naviculadealpina*
Valve length (µm)	11.5–28.5	12–30	15–40	14–45	30–50	19–40	26–86
Valve width (µm)	5–6	3.5–5.5	5–7	5.5–8	5.5–7	4–7	8–12
Stria density (in 10 µm)	12–13	15–21	10–11	8–10	10–12	11–13	8–10
Striae around central area	irregularly shortened	irregularly shortened	single shortened on either side	irregularly shortened	irregularly shortened	2–4 striae distinctly shortened on either side	irregularly shortened
Lineolae density (in 10 µm)	ca. 50	ca. 44–51	--	ca. 40	ca. 30	ca. 50	ca. 26 (LM)
Valve shape	smaller elliptic, larger linear-elliptic-lanceolate	elliptic-lanceolate	elliptic-lanceolate to linear-lanceolate	elliptic to lanceolate to linear-elliptic-lanceolate	linear- elliptic -lanceolate	linear-lanceolate	linear-lanceolate to almost elliptical
Central area	elliptic and small	very small and asymmetric	elliptic and very small	small	large, transversely rectangular to elliptical	broadly rectangular or transapically elliptic	almost symmetrical, transversally rectangular
Valve apices	narrowly rounded	cuneately rounded	obtusely rounded	obtusely rounded	narrowed to a wedge, obtusely- rounded, never protracted	gradually wedge-shaped, finally obtusely rounded	obtusely wedge-shaped
Raphe	filiform	weakly lateral; central pores very close together	weakly lateral, central pores very close	filiform	strongly radiate	filiform	weakly to strongly lateral, lying outside the median area towards the central pores
References	this study	Mertens et al. (2014)	Lange-Bertalot (2001)	Lange-Bertalot (2001)	Tsarenko et al. (2000)	Cantonati et al. (2016)	Lange-Bertalot (2001)

In relation to the three taxa mentioned in the introduction as having been found in Van Lake by Legler and Krasske (1940) – *N.cryptocephala*, *N.capitatoradiata* and *N.veneta* – *N.vansee* is easily distinguished in LM by the shape of its apices, which are rounded, while all the others have narrow, protracted apices. Molecular phylogeny, despite the limitations in the sampling of taxa, also concurs to discriminate *N.vansee*a sp. nov. from these three species (Fig. 8).

### ﻿Genetic polymorphisms and genome evolution

The organellar genomes, especially the plastomes, show some interesting features. For example, introns are not considered to be conserved genetic elements and are known to vary amongst isolates of a single species (e.g. Gastineau et al. (2021b)), but they were fully conserved between our two isolates, whereas protein-coding genes displayed non-silent polymorphisms. In addition, all three markers commonly used for phylogeny reconstruction in diatoms (18S, *rbcL* and *psbC*) exhibited single nucleotide polymorphisms in *N.vanseea*; this was especially surprising for the nuclear 18S gene, which generally exhibits very few differences between closely-related species (Evans et al. 2007). The polymorphism in *18S* appeared to be in the variable V2 region, while the V4 or V9 regions, which are often used in metabarcoding studies, were found fully conserved.

In the plastome of *N.vanseea*, the *ycf35* gene has seemingly been turned to a pseudogene, which would be the first time to our knowledge that this has been observed in diatoms, although *ycf35* pseudogenes have been observed in Rhodophyta (Costa et al. 2016). This gene seems to be lost altogether amongst other taxa, such as *Rhizosoleniaimbricata* Brightwell, 1858 (Sabir et al. 2014) or *Proboscia* sp. and *Licmophora* sp. (Yu et al. 2018). It is not clear if the gene has been completely lost in these species or if it has been transferred to the nuclear genome, which is known to have happened with the plastid *petF* gene in *Thalassiosiraoceanica* (Lommer et al. 2010). In *N.vanseea*, the *ycf35* gene is likely no longer functional. The size of the Ycf35 protein amongst diatoms is ca. 1130 AA long. Its origin can be traced back to Cyanobacteria. Its function is unknown, but it has been suggested, based on experiments conducted on *Synechocystis*, to participate in CO_2_ capture (Jiang et al. 2015). The results obtained from *N.vansee* sp. nov. suggest that it is not necessary to its metabolism and survival, unless *ycf35* is also already present in the nucleus.

Our study also illustrates the added value that next generation sequencing provides when describing new species, in three ways. First, it is a convenient way to gather data for multigene phylogenies, whatever the species considered. Second, in the current case with *N.vanseea*, it made it possible to find SNPs in supposedly conserved genes of two sympatric strains of the same species. This needs to be taken into account in interpreting phylogenetic and metabarcoding analyses. Third, serendipitous discoveries can occur that increase our knowledge of the organellar genomes of diatoms and other stramenopiles, such as the loss of a functional *ycf35* gene here or the first documented L1917 intron found in a stramenopile.

## Supplementary Material

XML Treatment for Navicula vanseea

## ﻿Additional information

### Author ORCIDs

Elif Yılmaz https://orcid.org/0000-0001-7939-1814

David G. Mann https://orcid.org/0000-0003-0522-6802

Romain Gastineau https://orcid.org/0000-0001-8661-5118

Rosa Trobajo https://orcid.org/0000-0001-9498-3797

Cüneyt Nadir Solak https://orcid.org/0000-0003-2334-4271

Ewa Górecka https://orcid.org/0000-0003-0590-7480

Monique Turmel https://orcid.org/0000-0001-7060-035X

Claude Lemieux https://orcid.org/0000-0001-9580-8042

Nesil Ertorun https://orcid.org/0000-0001-6224-7314

Andrzej Witkowski https://orcid.org/0000-0003-1714-218X

### Data availability

All sequences have been deposited and are already available on GenBank with accession numbers OR797293, OR797294, OR795084, OR795085, OR795086. The alignment, partition file and complete tree in Newick format can be obtained from Zenodo following this link: https://doi.org/10.5281/zenodo.10518939.
